# Deer Meat as the Source for a Sporadic Case of *Escherichia coli* O157:H7 Infection, Connecticut^1^

**DOI:** 10.3201/eid0805.010373

**Published:** 2002-05

**Authors:** Terry Rabatsky-Ehr, Douglas Dingman, Ruthanne Marcus, Robert Howard, Aristea Kinney, Patricia Mshar

**Affiliations:** * Connecticut Emerging Infections Program, New Haven, Connecticut, USA; † The Connecticut Agricultural Experiment Station, New Haven, Connecticut, USA; ‡ The Connecticut Department of Public Heath, Hartford, Connecticut, USA

**Keywords:** *Escherichia coli*,157:H7, foodborne illness, Pulsed-field gel electrophoresis, PFGE, White-tailed deer, venison, epidemiology, hunters, diarrhea, child

## Abstract

We report a case of *Escherichia coli* O157:H7, which was acquired by eating wild White-Tailed deer (*Odocoileus virginianus*). DNA fingerprint analysis verified venison as the source of infection. This pediatric case emphasizes the need for dissemination of information to hunters regarding the safe handling and processing of venison.

*Escherichia coli* O157:H7 (O157) is a cause of acute infectious diarrhea in humans and the leading cause of hemolytic uremic syndrome, especially among children in the United States (*1*). Many animals, including cattle, sheep, and goats, are known to harbor O157; however, cattle are most often implicated as the zoonotic source of human infection (*2*). Transmission is usually attributed to contaminated foods. Meats, other than beef, from which O157 has been isolated include pork, lamb, and poultry (*2*,*3*). Although several reports document the presence of O157 in deer (*4*–*6*), only one report (*4*) has shown evidence of an O157 infection from eating venison. This report was specific to the Black-Tailed deer (*Odocoileus hemionus*). To our knowledge, this is the first case of O157 infection linked with eating wild White-Tailed deer (*Odocoileus virginianus*) meat.

## Case Report

A previously healthy 7-year-old boy was seen at a Connecticut emergency room with a 3-day history of gastrointestinal illness. Symptoms included bloody diarrhea, abdominal cramps, and nausea. The child ate treated with antibiotics as an outpatient; diarrhea resolved after 6 days. The child’s stool sample was positive for O157. Stool samples were not obtained from other family members.

Two days before the child’s onset of illness, his father butchered and grilled freshly killed venison for the family. The child eat a large quantity of undercooked (red), gamey-tasting grilled venison tenderloin. His father ate a few bites of the venison; his mother and sister ate none. The only other family member to report symptoms of illness was the father, who reported having an “unsettled stomach” without diarrhea the same day as his son’s onset of illness. Four weeks later, O157 was recovered from a frozen sample of uncooked venison obtained from the same carcass as the fresh, grilled tenderloin.

As part of routine disease surveillance, all patients with O157 infections who are reported to the Connecticut Department of Public Health (CDPH) are interviewed by telephone, using a standardized questionnaire. Parents of the 7-year-old boy were interviewed 2 weeks after the onset of symptoms, and information about his clinical illness and potential exposures was collected. A second interview, conducted 2 weeks later, sought additional information on illness in other family members and on deer handling and processing practices. Permission to collect samples of uncooked deer meat stored in the family freezer was also obtained.

The O157 patient isolate was sent to the CDPH laboratory for confirmation, H antigen determination, and subtyping by DNA fingerprinting using pulsed-field gel electrophoresis (PFGE). The isolate was cultured on sorbitol-MacConkey agar; sorbitol negative colonies were identified as O157 by standard methods (*7*) and subtyped by PFGE as described by Barrett et al. (*8*).

Three separate packages of White-Tailed deer meat, frozen for 25 days, were obtained from the child’s family and processed at the Connecticut Agricultural Experiment Station. For all three packages (steak, butterfly cut, and sausage pieces), a combined weight of 25 g frozen meat shavings were macerated, incubated in enrichment medium, and immunomagnetically separated (IMS), according to the manufacturers’ instructions (Dynal, Inc., Lake Success, NY). Magnetic beads were washed during the IMS extraction procedure as reported by Tomoyasu (*9*). Final suspensions of the magnetic beads were plated on cefixime-tellurite sorbitol MacConkey agar; sorbitol-negative colonies were confirmed using API20E (bioMérieux Vitek, Inc., Hazelwood, MO), serotyped using the RIM *E. coli* O157:H7 latex test (Remel, Lenexa, KS), and subtyped by DNA fingerprinting using PFGE (*8*). To verify uniqueness and confirm indistinguishable PFGE patterns, repeat subtyping by PFGE of patient and venison isolates was done at the CDPH laboratory. Restriction-fragment banding patterns were matched digitally using a Gel doc 1000 System (Bio-Rad, Hercules, CA) and compared using the Molecular Analyst Plus software (Bio-Rad). Molecular Analyst Fingerprinting DST version 1.6 software (Bi0-Rad) for screening DNA patterns permitted a 3% molecular weight matching-tolerance; all matches were confirmed visually.

Both the clinical and venison O157 isolates were confirmed biochemically as *E. coli* and serotyped as O157:H7. DNA fingerprint analysis using PFGE demonstrated a pattern from the uncooked venison O157 isolate that was indistinguishable from the pattern of the clinical O157 isolate (Figure) and unique (occurring only once) among 26 patterns as previously described.

**Figure:**
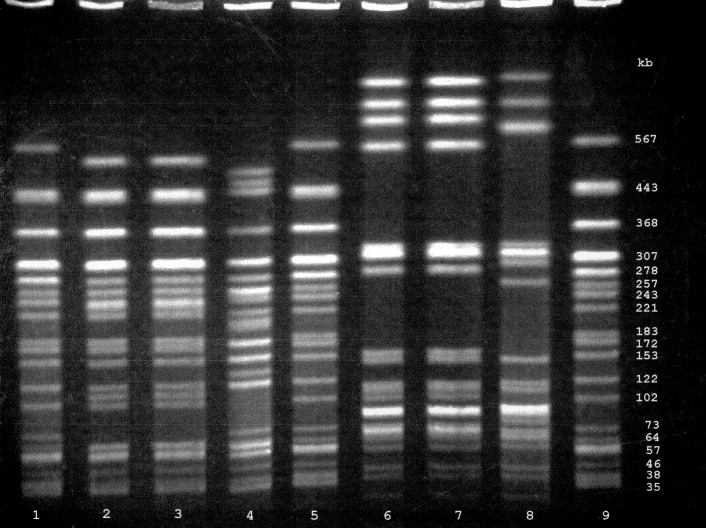
Pulsed-field gel electrophoresis of O157 isolates from the Connecticut child and the deer meat showing *Xba*I and *Bln*I-digested genomic DNA. Lanes 1, 5, and 9 are *Escherichia coli* G5244, a standard strain used to characterize molecular size; lanes 2 (*Xba*I) and 6 (*Bln*I) are digests from the child’s O157 isolate, lanes 3 (*Xba*I) and 7 (*Bln*I) are digests from the deer meat O157 isolate, and lanes 4 (*Xba*I) and 8 (*Bln*I) are digests from an unrelated O157 patient. Numbers at right are molecular sizes (in base pairs).

Interviews with the child’s parents found no history of traditional exposures for O157 infection. The child’s father provided information regarding the deer hunt and venison processing. The deer was shot (but not immediately killed) at noon in mid-November in Vermont. After tracking the wounded animal for 2 hours, the hunters located and field dressed the dead animal. An abdominal gunshot wound had resulted in intestinal rupture; no intact internal organs were visible when the deer was eviscerated in the field. No rinsing of the intestinal cavity occurred, as is general practice among deer hunters. The deer was dragged to a truck, brought back to camp, and hung outside overnight before being transported from southern Vermont to Connecticut. The deer was again hung outdoors overnight. Ambient air temperature ranged from 0°C-13°C during this period. The following morning the deer was skinned and cut into large sections. Individual sections were further cut, trimmed, and rinsed under running water before being packaged and stored in a home freezer. The tenderloin was rinsed, placed on a clean plate, refrigerated, and grilled outdoors that evening.

## Discussion

This investigation implicates venison from White-Tailed deer (*O. virginianus*) as the source of human O157 infection. We speculate that the deer acquired O157 from cows grazing on dairy farms in Vermont. The prevalence of O157 in White-Tailed deer sharing rangeland with cattle has been well documented (*4*–*6*). In addition, a field prevalence study in Georgia found that 3 (4%) of 77 hunter-killed White-Tailed deer carried O157 (*5*). Deer, like cattle, are transient carriers of O157 and are more likely to be colonized with O157 in the fall and winter (*6*). Thus, deer are most likely to carry O157 during the time of greatest human exposure, the fall hunting season.

An estimated 11.3 million Americans hunt big game such as deer or elk each year (*10*). Nationwide, the annual big game hunting prevalence rate is 7%; regional rates vary from a low of 4% in the Pacific States (Alaska, California, Hawaii, Oregon, and Washington) to a high of 14% in the North Central States (Kansas, Iowa, Minnesota, Missouri, Nebraska, North Dakota, and South Dakota). Relatively few cases of O157 infection have been associated with eating venison over the many years of deer hunting. In a 1995 report, a cluster of household cases was linked with eating jerky made from Black-Tailed deer meat, and a sporadic case in 1987 of O157 infection in Washington State was linked to venison (*4*).

Routine molecular subtyping of O157 isolates by the CDPH laboratory allowed us to link the sporadic case of O157 with eating venison. Fifty-five patient isolates were subtyped during that same year; 26 distinct patterns were identified. Twenty of these patterns (77%) were unique. This marked heterogeneity of isolates is not limited to Connecticut and emphasizes that many O157 infections are sporadic and caused by contamination of raw foods as well as food preparation and hygiene behaviors.

Multiple factors contributed to the contamination of the deer meat that was eaten by the Connecticut child. The abdominal gunshot wound increased the likelihood that intestinal contents initially contaminated the deer carcass. In addition, the extended time it took the deer to die, fecal contamination of the abdominal cavity, the warm day and mild evening temperatures, and the 2-day interval between deer kill and processing likely supported the dissemination and growth of O157 throughout the carcass. Lastly, a large quantity of undercooked venison tenderloin was eaten.

Hunters who handle wild game in the field are sometimes unaware of the risk of contaminating the meat with foodborne pathogens while dressing, handling, and transporting it. Contamination of game is usually related to the manner in which the animal is killed, dressed, handled, or processed. Improper temperature control, preservation, and cooking may also contribute to contaminated game. Proper handling of deer carcasses begins in the field with a clean shot to the neck or torso (lungs, heart, liver) and quick removal of the intestines/entrails (field dressing) from the abdominal cavity. If any of the internal organs smell offensive or exhibit discharge or blood in the muscle, the flesh is unfit for consumption. The abdominal cavity should be cleaned, dried, and cooled to <5°C until the meat is processed.

This case study provides direct evidence for O157 in White-Tailed deer and is the first report to link human illness to the presence of O157 in this species of deer. Our findings contribute to the body of evidence that eating venison may be a source of human infection and highlight the need to provide hunters with guidelines for the proper handling and processing of deer carcasses.
